# Psychometric properties of the Arabic generalized anxiety disorder scale (GAD-5) in a non-clinical sample of Arabic-speaking adults

**DOI:** 10.3389/fpsyg.2025.1582398

**Published:** 2025-07-08

**Authors:** Toni Sawma, Pio Abd El Nour, Diana Malaeb, Fouad Sakr, Mariam Dabbous, Sami El-Khatib, Feten Fekih-Romdhane, Souheil Hallit, Sahar Obeid

**Affiliations:** ^1^Department of Psychology and Education, School of Arts and Sciences, Lebanese American University, Jbeil, Lebanon; ^2^School of Medicine and Medical Sciences, Holy Spirit University of Kaslik, Jounieh, Lebanon; ^3^College of Pharmacy, Gulf Medical University, Ajman, United Arab Emirates; ^4^School of Pharmacy, Lebanese International University, Beirut, Lebanon; ^5^Department of Biomedical Sciences,School of Arts and Sciences, Lebanese International University, Bekaa, Lebanon; ^6^Center for Applied Mathematics and Bioinformatics (CAMB), Gulf University for Science and Technology (GUST), Hawally, Kuwait; ^7^Department of Psychiatry “Ibn Omrane”, The Tunisian Center of Early Intervention in Psychosis, Razi Hospital, Manouba, Tunisia; ^8^Faculty of Medicine of Tunis, Tunis El Manar University, Tunis, Tunisia; ^9^Department of Psychology, College of Humanities, Effat University, Jeddah, Saudi Arabia; ^10^Applied Science Research Center, Applied Science Private University, Amman, Jordan

**Keywords:** generalized anxiety disorder, psychometric properties, Arabic, Lebanon, GAD-5

## Abstract

**Background:**

generalized anxiety disorder (GAD) is a highly prevalent psychological disorder with a frequent distribution among the general population and in primary care configurations. GAD-5 is one of the many present scales with little research on, but has the potential to orient clinical decisions due to its unique features. Validating a simple and cost-effective tool to assess the GAD in the Arabic-speaking population, primarily residing in the Middle-East and North-Africa (MENA) region, would be highly beneficial. The study aimed to translate GAD-5 into Arabic and evaluate its psychometric properties, including internal reliability, sex invariance, composite reliability, and correlation with a measure of psychological distress.

**Methods:**

a total of 629 Arabic-speaking adults were recruited in May 2023. A self-administered anonymous survey was distributed through social media using a Google Forms link. We used the SPSS AMOS software v.28 to conduct the confirmatory factor analysis of the GAD-5 scale.

**Results:**

the fit indices deriving from the confirmatory factor analysis indicated that the one-factor model of the GAD items was acceptable. The convergent validity for this model and the internal consistency of the scale were good. Configural, metric, and scalar invariance was supported across sex. A higher mean anxiety score was seen in females compared to males. Higher GAD-5 scores were significantly associated with higher anxiety, depression and stress as measured by the Depression, Anxiety and Stress 8 items (DASS-8) scale, showing convergent and concurrent validity.

**Conclusion:**

our findings confirmed the briefness, validity and reliability of the Arabic version of the GAD-5 scale which support its employment as a screening tool in the general population. Considering these results, we advise using it for therapeutic and research motives among the Arabic-speaking individuals.

## Introduction

Generalized Anxiety Disorder (GAD) is a common mental health disorder characterized by persistent, excessive and unrealistic fear and worry about the multiple facets of life (family, health, finances, work or school; Stein et al., 2021; Leonard and Abramovitch, 2019). GAD is frequently distributed worldwide, reaching an estimated lifetime prevalence of 3.7% in adults of the general population, with females having approximately twice as higher rates when compared to males (Yang et al., 2021). Additionally, in the primary care setting, GAD is the most common anxiety disorder contracted, having an approximate 8% prevalence (Sapra et al., 2020). A Finnish study specified that the repartition of GAD among primary care patients was 4.1% for males and 7.1% for females (Kujanpää et al., 2014).

GAD can present clinically as a generalized worry concomitant with non-specific physical and psychological issues (DeMartini et al., 2019). There is also an intimate link between anxiety and other negative emotional states, particularly depression. While anxiety and depression have similar emotional profiles (Clark and Watson, 1991; Brady and Kendall, 1992), they are different in terms of several areas. Blumberg and Izard (1986) pointed out that while fear and apprehension are dominant in anxiety, sadness and lack of energy are central features of depression. Adding to that, stress, particularly in early life, has been shown to exert a significant influence on risk of anxiety as well as further mental disorders (e.g., depression; Bartlett et al., 2017; Slavich and Irwin, 2014). Additionally, patients with GAD may often present with somatic manifestations which can induce a diagnostic plight, resulting in a poor diagnosis of the disease (National Collaborating Centre for Mental Health, 2011). For this reason, efforts have been put in order to set up a GAD scale, which is a screening test that may be used in primary care settings to help orient suspicions of GAD and reduce confounding differentials.

There exists a variety of scales used by clinicians to evaluate anxiety disorders; the most prominent ones include: Hamilton Anxiety Rating Scale (HAM-A; Gunver et al., 2021), Beck Anxiety Inventory (BAI; Oh et al., 2018), Penn State Worry Questionnaire (PSWQ; Johnco et al., 2022), State-Trait Anxiety Inventory (STAI; Knowles and Olatunji, 2020), and finally the widely used GAD scale with its three evolutionary faces (GAD-9, GAD-7, and GAD-5). Before detailing the series of the GAD scales, we would like to point out that of all those present scales, the GAD scale has shown to be the most effective and the most clinically-friendly screening test for GAD due to its brief, quick and precise structure (Dhira et al., 2021), which opposes the non-specific broader features and the lengthy set of items of the other tests.

### GAD scale

The GAD scale initially begun as nine items summarizing all of the Diagnostic and Statistical Manual of Mental Disorders (DSM-IV) diagnostic criteria for GAD, in addition to four items deduced from existing anxiety scales (Spitzer et al., 2006). Afterwards, a Generalized Anxiety Disorder-7 scale (GAD-7) is a seven-item model of the initial scale that appeared, and has demonstrated high sensitivity and specificity in both the general population and primary care patients (Moreno et al., 2019). As for the worldwide authentication of the GAD-7 scale, research has proved both unidimensional structure and internal consistency in mostly university students across, Lithuania (Pranckeviciene et al., 2022), China (Gong et al., 2021), Russia (Zinchuk et al., 2021), Spain (Martínez-Vázquez et al., 2022), Portugal (Bártolo et al., 2017), and the United States (Byrd-Bredbenner et al., 2021). In Latin America, six countries (Peru, Ecuador, Argentina, Bolivia, Chile and Columbia) demonstrated the scale's consistency (López et al., 2025). Subsequently, the Generalized Anxiety Disorder-5 (GAD-5) scale is a five-item version which is directly correlated with the International Classification of Diseases (ICD-11) diagnostic guidelines for anxiety and depression, and was obtained from several studies of the primary care population (Goldberg et al., 2017). The simplicity and briefness of the GAD-5 scale provide it a favorable condition to be more potentially applied in the general population and in primary care settings than the GAD-7 and the GAD-9 scales (Goldberg et al., 2017).

Consequently, a unique validation study conducted in Mexico conveyed the unidimensional structure as well as the reliability of the GAD-5 scale and noted the presence of configural and metric invariance in the comparison by sex, age, and educational level, as well as scalar invariance in the comparison by sex and age (Astudillo-García et al., 2022). To our knowledge, the validation of the GAD-5 scale and measurement of its parameters' invariances has only been done across the Mexican population. Validating the invariance of parameters across ages, educational levels and sexes aids in understanding whether the five measures of the GAD-5 scale are equivalent throughout different socio-demographic groups (Astudillo-García et al., 2022). Hence, since the Arabic version of the GAD-5 addresses a major linguistic barrier in psychological research, its validation is highly significant.

### The present study

In Lebanon, a study done in 2009 proved that Lebanon expressed a 12 months rate of 11.2% for any anxiety disorder (Tanios et al., 2009). Despite the lack of recent data and numbers, it is theoretically assumed that anxiety disorders are currently more routinely encountered in the Lebanese population especially after the year 2019 which was a hallmark that historically altered the life of the Lebanese citizens due to successive events such as COVID-19, the long-lasting economic crisis, as well as the Beirut explosion which had detrimental outcomes on the general population (Hashim et al., 2022); however, no research was done to solely evaluate GAD in Lebanon using the GAD-5 scale, presumably due to the unavailability of an Arabic language-validated scale. Therefore, this study's goal was to investigate the psychometric qualities of an Arabic translation of the GAD-5 in a group of adult's Lebanese participants. The Arabic GAD-5 is expected to: (1) replicate the one factor structure that was first proposed; (2) exhibit strong composite validity and invariance of measurements by sex (males vs. females), age and educational level; and (3) show sufficient patterns of correlations with depression and stress.

## Methods

### Study design

A survey was meticulously designed using Google forms and disseminated through various digital platforms, including messaging applications and social media networks; in a snowball sampling strategy. The study engaged a cohort of 629 participants, who were recruited in May 2023. The eligibility criteria encompassed: (1) being a resident and citizen of Lebanon, (2) attaining the age of 18 years or older, and (3) possessing internet access. Participants unwilling to complete the questionnaire were excluded. Upon granting digital informed consent, participants were prompted to undertake the instruments detailed below. The survey was conducted under the principles of anonymity, with participants engaging voluntarily and without any form of remuneration. On average, the completion of the survey spanned 20 min.

We aimed to enroll a minimum of 120 adolescents following the recommendations of Mundfrom et al. of 3 to 20 times the number of the scale's variables (Mundfrom et al., 2005).

### Questionnaire

In our research, we held the principles of confidentiality in the highest regard. Our comprehensive study explored sociodemographic variables including age, sex, marital status, and educational level. The Household Crowding Index, which serves as an indicator of the family's socioeconomic status (Melki et al., 2004), was calculated as the ratio of the total number of individuals residing in the household to the total number of rooms within the dwelling (excluding kitchens and bathrooms).

The survey also included the *Generalized Anxiety Disorder-5 (GAD-5)*, a 5-item scale evaluating anxiety symptoms experienced in the last 2 weeks (Goldberg et al., 2017). Participants evaluated how well each statement described their recent experiences on a 10-point Likert scale (“0 = does not describe me and 10 = describes me exactly). Higher scores indicate higher GAD.

*Depression Anxiety Stress Scale- 8 items (DASS-8)*. This scale is a shortened version of the Depression, Anxiety and Stress Scales-21 (DASS-21; Ali et al., 2022). Validated in Arabic (Ali et al., 2024), this scale is composed of 8 items rated on a Likert scale (0 = never to 3 = always). Higher scores reflect more psychological distress.

### Translation procedure

The translation of the GAD-5 from English to Arabic was meticulously executed by a mental health professional, followed by a reverse translation from Arabic to English by a certified linguist. Subsequent to these translations, a comprehensive comparison of the English versions was conducted by the translators to ensure consistency and equivalence in the conveyed meaning of the statements. Furthermore, a pilot study involving 30 individuals was carried out to validate the clarity and comprehensibility of the questions. This preliminary phase showed no disparities between the two versions.

### Statistical analysis

There were no missing responses in the dataset. We used data from the total sample to conduct a CFA using the SPSS AMOS v.30 software. Parameter estimates were obtained using the maximum likelihood method. Multiple fit indices were calculated: root mean square error of approximation (RMSEA; ≤ 0.08), standardized root mean square residual (SRMR; ≤ 0.05), Tucker-Lewis Index (TLI; ≥0.90), Comparative Fit Index (CFI; ≥0.90), Goodness of Fit Index (GFI; ≥0.90), Adjusted Goodness of Fit Index (AGFI; ≥0.90), and Normed Fit Index (NFI; ≥0.90; Hu and Bentler, 1999). Additionally, values of the average variance extracted (AVE) ≥0.50 indicated evidence of convergent validity (Malhotra and Dash, 2011). Multivariate normality was not verified at first (Bollen-Stine bootstrap *p* = 0.042); therefore, we performed non-parametric bootstrapping procedure.

To examine sex invariance of GAD-5 scores, we conducted multi-group CFA (Chen, 2007) using the total sample. Measurement invariance was assessed at the configural, metric, and scalar levels (Vadenberg and Lance, 2000). We accepted ΔCFI ≤ 0.010 and ΔRMSEA ≤ 0.015 or ΔSRMR ≤ 0.010 as evidence of invariance (Chen, 2007).

Composite reliability was assessed using McDonald's ω and Cronbach's α, with values greater than 0.70 reflecting adequate composite reliability. The GAD-5 scores were considered normally distributed as shown by the skewness (=0.684) and kurtosis (−0.346) values, varying between −1 and +1 (Hair Jr et al., 2021). Pearson test was used to correlate the GAD-5 scores with the other scales in the survey. The Student *t* test was used to compare two means. Values ≤ 0.10 were considered weak, ~0.30 were considered moderate, and ~0.50 were considered strong correlations (Cohen, 1992).

## Results

### Sociodemographic and other characteristics of the sample

Six hundred twenty-nine participants participated in this study, with a mean age of 29.11 ± 12.22 years (min = 18; max = 70) and 70.9% females (Table 1).

**Table 1:** Sociodemographic and other characteristics of the sample (*N* = 629).

**Variable**	***N* (%)**
**Sex**	
Male	183 (29.1%)
Female	446 (70.9%)
**Education**	
Secondary or less	169 (26.9%)
University	460 (73.1%)
**Mean** ±**SD**	
Age (years)	29.11 ± 12.22
Household crowding index (persons/room)	1.09 ± .52
DASS-8 depression	3.31 ± 2.44
DASS-8 anxiety	3.22 ± 2.52
DASS-8 stress	2.60 ± 1.66
GAD-5 anxiety	15.54 ± 12.19

DASS-8, depression, anxiety and stress 8 items scale; GAD, generalized anxiety disorder.

### Confirmatory factor analysis

**Figure 1:**
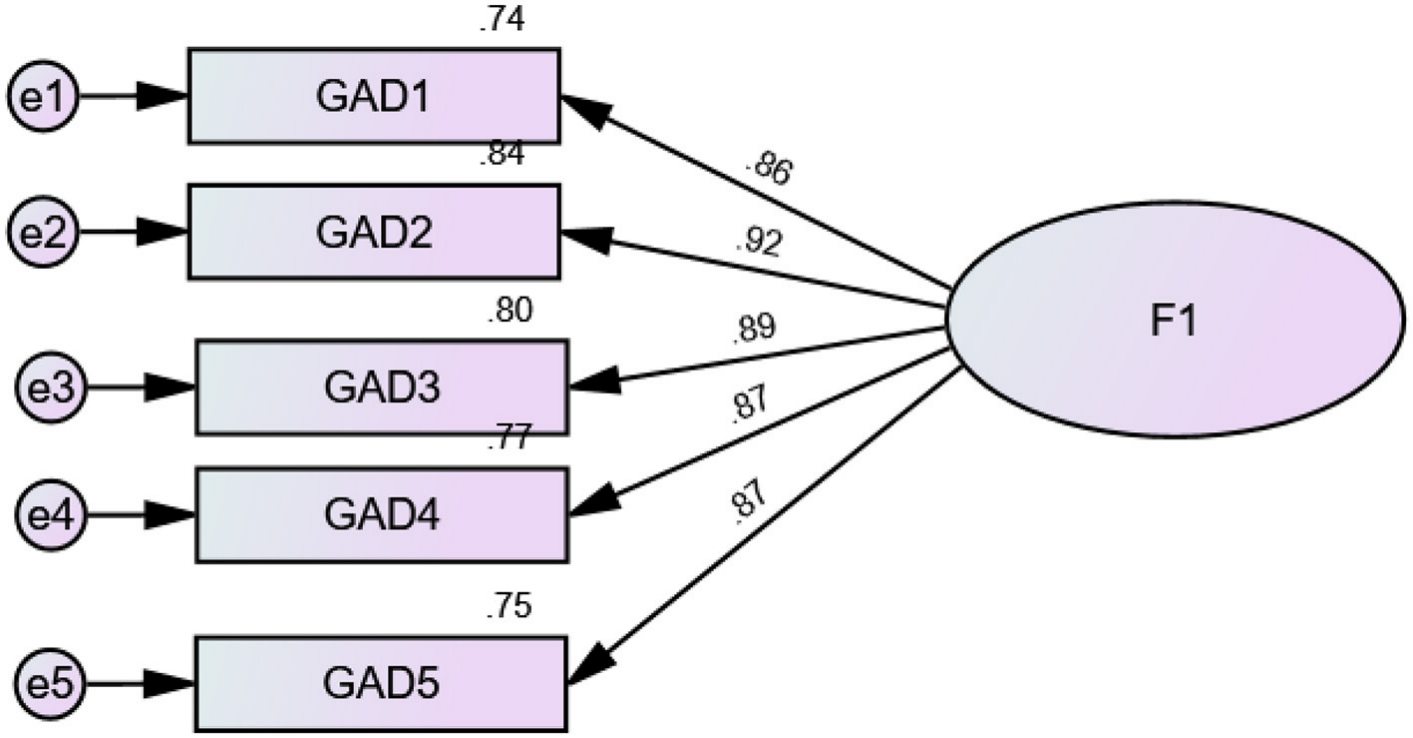
Standardized estimates of factor loadings of the generalized anxiety disorder 5 items scale deriving from the confirmatory factor analysis.

CFA indicated that fit of the one-factor model of the GAD items was acceptable: RMSEA = 0.100 (90% CI 0.071, 0.131), SRMR = 0.014, CFI = 0.989, TLI = 0.979, GFI = 0.979, AGFI = 0.937, NFI = 0.988. The standardized estimates of factor loadings were all adequate (Figure 1). The convergent validity for this model was adequate, as AVE = 0.78. The internal consistency of the scale was excellent (ω = 0.95/α = 0.95).

### Sex invariance

All indices suggested that configural, metric, and scalar invariance was supported across sex (Table 2). The results showed that a higher mean GAD-5 scores was seen in females (*M* = 16.16, *SD* = 12.63) compared to males (*M* = 14.03, *SD* = 10.93), *t*_(627)_ = −2.12, *p* = 0.035. No significant difference was found between participants with a university level of education (*M* = 15.97, *SD* = 12.53) vs. secondary or less (*M* = 14.37, *SD* = 11.15), *t*_(627)_ = −1.54, *p* = 0.125. No significant difference was found between participants in terms of age (18–25 years: *M* = 15.99, SD = 11.91; 26–35 years: *M* = 16.19, SD = 13.62; 36–59 years: M = 14.13, SD = 11.81; 60+ years: *M* = 11.90, SD = 8.56; *F*_(3, 625)_ = 1.45; *p* = 0.226).

**Table 2:** Measurement Invariance in the total sample.

**Model**	**CFI**	**RMSEA**	**SRMR**	**Model comparison**	**ΔCFI**	**ΔRMSEA**	**ΔSRMR**
**Model 1: across sex**							
Configural	0.983	0.089	0.015				
Metric	0.982	0.077	0.019	Configural vs. metric	0.001	0.012	0.004
Scalar	0.981	0.069	0.020	Metric vs. scalar	0.001	0.008	0.001
**Model 2: across education**							
Configural	0.978	0.103	0.029				
Metric	0.976	0.091	0.033	Configural vs. metric	0.002	0.012	0.004
Scalar	0.976	0.077	0.033	Metric vs. scalar	<0.001	0.014	<0.001
**Model 3: across age**							
Configural	0.975	0.078	0.023				
Metric	0.975	0.062	0.024	Configural vs. metric	<0.001	0.016	0.001
Scalar	0.972	0.056	0.024	Metric vs. scalar	0.003	0.006	<0.001

CFI, comparative fit index; RMSEA, root mean square error of approximation; SRMR, standardized root mean square residual.

### Convergent validity

Higher GAD-5 scores were significantly and moderately associated with higher anxiety (*r* = 0.47; *p* < 0.001), depression (*r* = 0.47; *p* < 0.001) and stress (*r* = 0.54; *p* < 0.001) as measured by the DASS-8 scale.

### Discriminant validity

Since the square root of the AVE (=0.88) is higher than the correlations of the GAD-5 score with the other scores, therefore, we assumed that discriminant validity was confirmed.

## Discussion

By involving a sample size extracted from Lebanese Arabic-speaking individuals which provides a sufficient statistical power, we aimed to validate the Arabic version of the GAD-5 scale as well as to evaluate measurement invariance through three sociodemographic factors: age, sex and educational level.

Our CFA demonstrated that the GAD-5 scale model abides by a one factor structure as initially proposed by the Mexican study (Astudillo-García et al., 2022), meaning that all the five items provided in the test are believed to measure a single underlying construct or factor. This implies that the variability in responses to all five items can be explained by one common factor, and that all the present items are assumed to measure the generalized anxiety disorder. Furthermore, although the RMSEA values exceeded the recommended threshold of 0.08, RMSEA tends to be inflated in models with low degrees of freedom (*df* < 50; Kenny and McCoach, 2003; Kenny et al., 2015). Given that our models have low degrees of freedom (*df* = 5), RMSEA may not be a reliable indicator in this case. Instead, model evaluation should rely on the other fit indices such as CFI and SRMR, which have been shown to be more stable under these conditions (Lai and Green, 2016). In our study, both CFI and SRMR values were adequate, supporting a good fit despite the elevated RMSEA. Additionally, our results indicate that the GAD-5 scale showed excellent internal reliability, with both Cronbach's Alpha and McDonald's Omega coefficients at 0.95. These results suggest that the one-factor model adequately represents the underlying structure of the scale. All standardized factor loadings were substantial, showing that each item contributed meaningfully to the latent construct of generalized anxiety. Furthermore, the model demonstrated strong convergent validity, as shown by an average variance extracted (AVE) of 0.78. These findings underscore the high reliability of the current Arabic version and highlight the need for further research to explore cross-cultural variations in this measure's internal consistency.

Moreover, our analysis indicates that configural, metric, and scalar invariance are upheld across sex, age as well as educational level seen by ΔCFI <0.01; ΔRMSEA <0.05; ΔSRMR <0.05 consistently across sex, age and educational level and in both configural vs. metric as well as metric vs. scalar analyses. In the Mexican validation, the results showed that the GAD-5 presents configural and metric invariance for sex, age, and educational level, and scalar invariance for sex and age groups (Astudillo-García et al., 2022). This proves that GAD-5, when converted to the Arabic version acts as an adequate screening tool and can be used in the general population to compare between men and women as well as educational levels, and can be utilized regardless of sex, age or educational level.

These findings stay consistent with the use of GAD-5 by individuals with different sociodemographic data despite the alternating structure of anxiety that is strongly dependent on sex, age as well as educational level. Anxiety is known to be higher in females than males (Torrano et al., 2020), varies across age, with adolescents, young and late adulthood individuals experiencing higher levels than middle adulthood people (Torrano et al., 2020; Tetzner and Schuth, 2016). In addition, individuals with lower educational levels tend to report increased anxiety levels when compared to individuals with higher educational attainment (Tetzner and Schuth, 2016). In the present study, no significant differences were found in GAD-5 scores across age groups or educational levels. Although this finding contrasts with previous literature, it may reflect the impact of Lebanon's ongoing socioeconomic challenges and recent adverse events, as outlined in the introduction section. These shared stressors may contribute to similarly high levels of anxiety across the population, regardless of age or educational attainment.

As for the convergent validity of the GAD-5 scale, it was supported by its significant and moderate correlations with related constructs measured by the DASS-8. Specifically, higher GAD-5 scores were positively associated with higher levels of anxiety (*r* = 0.47, *p* < 0.001), depression (*r* = 0.47, *p* < 0.001), and stress (*r* = 0.54, *p* < 0.001). These findings align with theoretical expectations, as generalized anxiety is known to co-occur with symptoms of depression and stress. The strength and significance of these associations provide further evidence that the GAD-5 effectively captures the broader emotional distress often experienced in individuals with anxiety, thereby supporting its convergent validity.

As previously mentioned, our findings supported notions present in the current literature stating that anxiety often coexists with depression as well as stress such that when anxiety levels escalate, stress and depression levels are exacerbated (Lei et al., 2020). Thus, the high correlation of the results between the GAD-5 scale and other existing scales as well as the current literature also confirms the scale's elevated concurrent validity.

### Clinical implications

Altogether, this study confers to the GAD-5 scale an elevated precision, accuracy, reliability as well as internal validity. These results potentiate important ramifications for the GAD-5 scale to be used in the Arab general population by both clinicians and researchers, advocating for a holistic approach to patient care, since this instrument works for both sexes, all ages as well as educational levels without the need for specific sociodemographic adjustments. Moreover, the fact that the GAD-5 scale is available in Arabic makes cross-cultural comparisons easier.

### Limitations

We should take into consideration some limitations as we analyze our study. The sample was recruited using the method of snowball sampling, so that generalization of findings may be limited. Information bias is present like all studies with a cross-sectional design. In addition, some important psychometric properties were not investigated in this study, such as inter-rater and test-retest reliability. As this study was conducted in Lebanon exclusively, more studies are warranted to explore whether the Arabic GAD-5 can be applied to Arabic-speaking adolescents from other Arab countries of different social and cultural backgrounds (such as Gulf or North African Arab countries).

## Conclusion

This study is the first in the Middle East to delve into the properties of the GAD-5 scale. Overall, the concise framework concurrent with the excellent reliability and briefness of our GAD-5 scale model that we demonstrated in this study confer a solid matrix for the utilization of this scale among Arabic-speaking adults. Given that it is a simple and safe tool with a high diagnostic value, researchers and clinicians each in his own environment can put this scale into use to contribute in their successive fields. This reliable and valid Arabic version of the GAD-5 scale could initiate a new era of research and potentially fill the gaps in the fields of psychology and psychiatry in the Arab world.

## Data Availability

The original contributions presented in the study are included in the article/supplementary material, further inquiries can be directed to the corresponding author.
